# A Case of Actinomycosis of the Nasopharynx

**DOI:** 10.7759/cureus.45710

**Published:** 2023-09-21

**Authors:** Dinie Tumaisuri, Farhana A, Farid Razali, Salina Husain

**Affiliations:** 1 Department of Otorhinolaryngology, Hospital Shah Alam Selangor, Shah Alam, MYS; 2 Department of Otorhinolaryngology, University Kebangsaan Malaysia Medical Center, Kuala Lumpur, MYS

**Keywords:** nose, nasopharyngoscopy, histopathological examination, nasopharynx, actinomycosis

## Abstract

Actinomycosis is a progressive granulomatous infection caused by the bacteria *Actinomyces israelii*. Classically, the three most common clinical forms are cervicofacial, thoracic, and abdominopelvic. On the contrary, nasopharyngeal actinomycosis is considered to be a rare clinical disease, and its occurrence is extremely low. The infection can take place without any preceding infection or on immunocompromised status. A 25-year-old male with no previous medical history presented with persistent nasal congestion and rhinorrhea. A nasal endoscopy examination revealed an unclearly demarcated nasopharyngeal mass, and a complete microbiology and pathology analysis showed actinomycetes colonies. After two weeks of oral Augmentin therapy, the patient's illness was completely eradicated. Diagnosis of nasopharyngeal actinomycosis is exceptionally crucial, and with early treatment of appropriate antibiotic therapy, the prognosis is excellent. Careful follow-up after adequate treatment as the possibility of frequent relapse is common.

## Introduction

Actinomycosis is a non-contagious chronic infection that is typically caused by filamentous, branching, gram-positive, anaerobic-to-microaerophilic bacteria, *Actinomyces israelii* [1]. They are distinctive predominant bacterial normal flora in the oral cavity. In most cases, actinomycosis are opportunistic infections; they can occur as a result of nasal trauma, surgical manipulation, contact with foreign bodies, or other trauma. The cervicofacial form of actinomycosis accounts for around 60% of cases. Studies on actinomycosis in the nasopharynx are rare [2]. Therefore, it is advised to assess with caution when treating a patient with inconclusive symptoms. In this report, we describe a rather exceptional and uncommon case. The images, microbiological results, and clinical course were also shown.

## Case presentation

**Figure 1:**
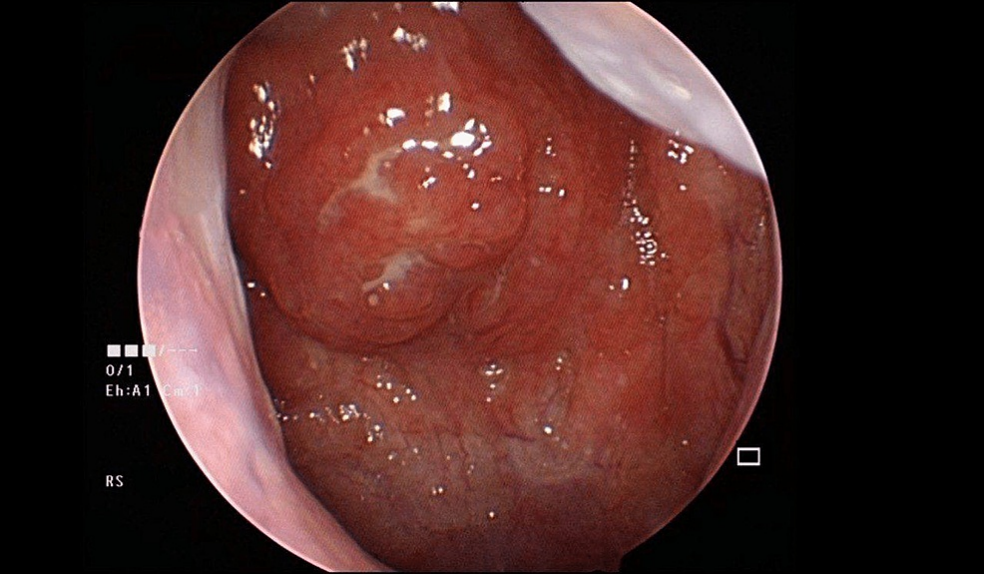
Nasopharyngoscopy examination revealing inflamed lymphoid hyperplasia originating from the nasopharynx

A 25-year-old male with a one-year history of persistent rhinorrhea and nasal congestion was referred to the otorhinolaryngology department of Hospital Shah Alam Selangor. He had an unremarkable past medical history. A thorough history revealed that there are no other complaints in the four main categories (ear, nose, neck) and others that may be explained by cranial nerve involvement. The clinical evaluation by direct nasopharyngoscopy with a rigid telescope showed a suspicious-looking unilateral inflamed lymphoid hyperplasia originating from the left posterior wall of the nasopharynx (Figure 1). Taking into account that he comes from a generation of a family of Chinese, therefore, a biopsy was carried out. The histopathology revealed basophilic radiating filaments within lymphoid stroma associated with *Actinomyces* colonies. For a duration of two weeks, systemic oral Augmentin 625 mg three times daily was administered in addition to alkaline nasal douche, nasal spray budesonide, and oral loratadine.

**Figure 2:**
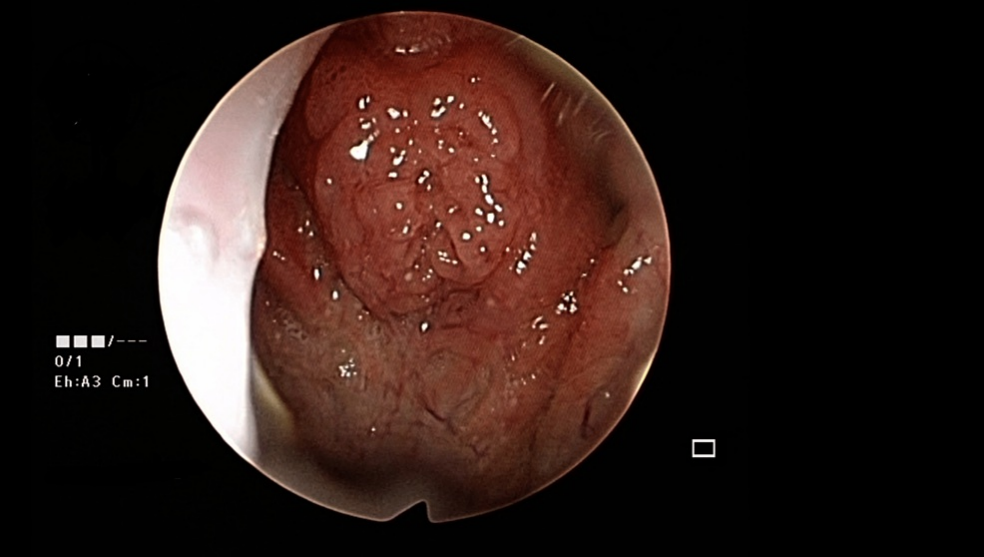
After two weeks of oral Augmentin, a nasopharyngoscopy revealed that the Actinomyces infection had resolved

This case was discussed with an infectious disease physician, who recommended repeating the biopsy and starting six weeks of IV C-penicillin or amoxicillin if the results revealed persistent *Actinomyces* infection. Tuberculosis screening had been performed. A rigid endoscope was used to thoroughly examine the nasopharynx; the adenoid tissue was less hypertrophic and not inflamed. A repeated biopsy and histopathological examination were performed, which revealed no *Actinomyces*. The recovery was uneventful, with no signs of recurrence in the following year (Figure 2).

## Discussion

Actinomycosis is a rare invasive bacterial disease in which 70% of the infections are due to *Actinomyces Israelii* [2]. *Actinomyces *contribute to the normal flora of the oral cavity but are less prominent in the GI tract and female urogenital tract. Because these microorganisms are opportunistic and not virulent, they are triggered by mucous membrane trauma or systemic immunodeficiency but can occur without any prior infections or backgrounds.

The incidence of the disease is greater in males aged 20-50 years [3]. Young adult males are commonly affected, which is attributed to a preponderance of males being involved in accidents and fights, causing soft tissue trauma and, thus, inoculation of bacteria. The reported male-to-female ratio is 3:1 [3].

The most distinct clinical forms of actinomycosis are cervicofacial, thoracic, and abdominal. Cervicofacial actinomycosis comprises 50-60% of all reported cases.

In response to the breaching of mucosal barriers and subsequent infection, the human body initiates a suppurative and granulomatous inflammatory response. This infection frequently ignores tissue planes and spreads contiguously to surrounding tissues and organs. Patients may present with painful multiple abscesses or persistent asymptomatic soft-tissue swelling and over time can lead to the formation of sinus tracts that discharge sulfur granules [4].

Previous reports demonstrated a two-step actinomycosis pathophysiology. First, a lesion has traumatized the mucosal lining of the area. According to some studies, nasal suction during nasal surgery causes damage to the nasopharynx that can lead to the development of nasopharyngeal actinomycosis. Second, another organism provides a synergistic anaerobic environment in which actinomycosis can proliferate [5].

Tissue biopsy culture with anaerobic pus cultures is the gold-standard test [1-4]. This is partially because *Actinomyces *are anaerobic or microaerophilic. Systemic antibiotics are often prescribed before diagnosis, which may reduce the identification rate. Histologically, actinomycosis shows chronic inflammation of granulomatous fibrous tissue, within which eosinophilic club-shaped structures are found surrounding a hematoxylin ophilic center. Consequently, the combination of microbiological analysis, clinical images, and histological findings should provide an accurate diagnosis of actinomycosis.

Most of the cases involve long durations of antibiotic treatment, and surgery can be adjunctive. Medical management in the form of antibiotics, including high-dose penicillin, is the antibiotic of choice. The duration may be variable from two to six weeks but can be shortened if surgical resection of the infected site has been performed [2].

Reportedly, the complications of nasopharyngeal actinomycosis resulting in a draining fistula may cause otitis media with effusion, nasal obstruction, and internal carotid artery occlusion as it has the ability to spread into adjacent tissue regardless of facial or anatomical barriers [3].

Outcome and mortality have improved with a high level of clinical suspicion when treating patients by diagnosing at an early stage and treating with appropriate antibiotic therapy. For extensive and complicated actinomycotic disease, aggressive antibiotics and surgical therapy are required [6].

## Conclusions

Nasopharyngeal actinomycosis is a rare occurrence. It arises in soft tissue and can mimic malignant tumors, making diagnosis difficult. In most cases, nasopharyngeal carcinoma was suspected in patients prior to diagnosis. Early treatment of actinomycosis with adequate antibiotic therapy promotes rapid recovery, resulting in a great prognosis. Patients may require extensive (six-week) treatment with high doses of penicillin or amoxicillin, but short-term antibiotic treatment can be appropriate. Further research should be done to offer optimal time periods for antibiotic treatment, as well as more novel information about the clinical evaluation and therapy of nasopharyngeal actinomycosis. Careful and consistent follow-up, in conjunction with local and imaging results, is critical for preventing recurrence.
